# Strategic roles for behaviour change communication in a changing malaria landscape

**DOI:** 10.1186/1475-2875-13-1

**Published:** 2014-01-02

**Authors:** Hannah Koenker, Joseph Keating, Martin Alilio, Angela Acosta, Matthew Lynch, Fatoumata Nafo-Traore

**Affiliations:** 1 Johns Hopkins Bloomberg School of Public Health, Center for Communication Programs, 111 Market Place Suite 310, Baltimore, MD 21202, USA; 2 Center for Applied Malaria Research and Evaluation, Tulane University School of Public Health and Tropical Medicine, 1440 Canal Street, Suite 2200, New Orleans, Louisiana 70112, USA; 3 United States Agency for International Development, Washington, District of Columbia, USA; 4 Roll Back Malaria Partnership, Geneva, Switzerland

**Keywords:** Malaria, Elimination, Behaviour change communication

## Abstract

Strong evidence suggests that quality strategic behaviour change communication (BCC) can improve malaria prevention and treatment behaviours. As progress is made towards malaria elimination, BCC becomes an even more important tool. BCC can be used 1) to reach populations who remain at risk as transmission dynamics change (e.g. mobile populations), 2) to facilitate identification of people with asymptomatic infections and their compliance with treatment, 3) to inform communities of the optimal timing of malaria control interventions, and 4) to explain changing diagnostic concerns (e.g. increasing false negatives as parasite density and multiplicity of infections fall) and treatment guidelines. The purpose of this commentary is to highlight the benefits and value for money that BCC brings to all aspects of malaria control, and to discuss areas of operations research needed as transmission dynamics change.

## 

Strategic use of behaviour change communication (BCC) applies targeted messages and tailored approaches to promote healthy behaviours and reduced risk taking. BCC, also known as social and behaviour change communication, encompasses health communication, social and community mobilization, and it evolved from information, education and communication (IEC) strategies. With components ranging from interpersonal communication between a community health worker and her client to multi-level mass media campaigns, evidence-based and theory-driven BCC interventions are an integral part of all types of health promotion and disease prevention, and have been shown to significantly improve behaviours, notably in the areas of family planning and HIV prevention [1-7], but also in hygiene and sanitation [8,9], nutrition [10,11], and other disease areas [12-14]. Strategically targeting messages and approaches allows BCC to focus on specific individuals, household, or communities to maximize results of health interventions. This results-based approach to control and prevention has been used in a variety of settings to assess or change behaviour related to malaria, and strong evidence suggests that quality BCC can improve malaria prevention and treatment behaviours [15-19]. This commentary highlights the benefits and value for money that BCC offers for malaria prevention, treatment and control, given the changing malaria transmission dynamics, and discusses potential areas for operations research.

Behavioural barriers to malaria control are well-documented: inconsistent or non-use of bed nets; delays in seeking effective treatment; and the distribution of intermittent preventive therapy (IPTp) without fully explaining its use to pregnant women, are but a few [20-29]. Adding BCC components to key malaria interventions can help individuals and communities overcome these barriers. BCC also complements the procurement and distribution of malaria commodities, such as long-lasting insecticidal nets (LLINs), rapid diagnostic tests (RDTs), artemisinin-combination therapy (ACT), insecticide for indoor residual spraying (IRS), and drugs for intermittent preventive treatment for pregnant women (IPTp), by ensuring that these commodities are accessed and then used appropriately at the right time, thus protecting investments.

## BCC for malaria control increases benefits and value for money

BCC increases the likelihood of a good return on investment for malaria programmes. It increases the likelihood that nets are used, ACT and SP are not wasted, and that IRS programmes reach their target coverage levels. BCC is used in malaria control to encourage families to hang and use their nets regularly, care for them and repair them when they’re torn, or to create demand for replacing nets on a continuous basis or as part of distribution campaigns. Another key role is informing and mobilizing communities to work with IRS spray teams, to follow instructions during and after spraying, and then promote continued use of LLINs following spraying. Adoption of diagnostic testing of fevers by both consumers and providers is a necessary step for improved treatment and surveillance of malaria, and is critical for the success of the Test, Treat and Track (T3) initiative [30]. BCC is also vital for creating demand for testing and to build trust in results, particularly when patients receive malaria-negative results and are unsure of what to do next. As malaria transmission dynamics change, malaria will cease to be the primary cause of fever and there is an urgent need to improve provider skills in communicating with and counselling patients. Communication campaigns that use interpersonal communication are recommended to improve treatment adherence and demand for and recognition of quality drugs [25,31,32]. BCC promotes ANC attendance and IPTp uptake [33], and training in interpersonal communication is critical for improving the quality of care providers give pregnant women [34].

As countries scale up malaria control and the epidemiology of malaria changes, many areas are seeing decreased transmission and progress toward elimination [35,36]. BCC continues to play important roles when transmission declines. For example, BCC promotes testing and treatment in hotspot areas within Zanzibar, and net and prophylaxis use for travellers, such as in Swaziland [37]. BCC can encourage protective behaviours such as net use even when the risk of malaria is greatly diminished. To support malaria elimination, so-called “hot-pops,” reservoirs of infection often not considered when planning control (often adult men, those that work at night, and travellers to endemic areas) will need to also be the focus of interventions, as described in a recent editorial in *The Lancet*[38]. BCC is likely to be crucial for convincing asymptomatic individuals that testing and treatment will help them as well as their communities, and for informing communities about changes in the optimal timing of interventions and the use of new occupation-based vector-control products.

## Investment also needed in malaria BCC research

Evidence for malaria BCC effectiveness is growing, but more high-quality data is needed, especially as transmission dynamics change. BCC is a proven intervention, although the bulk of robust evaluations of BCC to date have been focused on other health areas, including family planning and HIV [1-3,5,7,14]. Meta-analyses for family planning and HIV, for example, demonstrate that targeted behaviours are higher on average among those exposed to mass media interventions [14,39]. Recent analyses using propensity score matching (which simulates experimental designs and produces valid causal inferences using cross-sectional datasets) have shown that net use is 10–15 percentage points higher among those exposed to malaria messages, controlling for all other factors (Boulay M, unpublished data) [19]. It is clear that BCC interventions are most effective when a combination of approaches is used, weaving together mass media, interpersonal communication and structural approaches to promote new or modified behaviours [12,40]. In addition, hearing information from trusted sources has significant effects on behaviour; when combined with evidence of social norms promoted through mass media, these behaviours and attitudes are reinforced [40].

Solid planning must inform BCC interventions so that messages are targeted to key audiences, activities are founded on behavioural theories and formative research, and enough commodities are available to meet the demand generated in the population. The RBM Strategic Framework for Malaria Communication at the Country Level 2012–2017 lays out key steps for evidence-based, strategic behaviour change communication interventions [41]. As countries progress towards eliminating malaria, BCC strategies will need to be updated and adapted as transmission dynamics change and perception of risk is reduced.

Additional data is needed on the effectiveness of BCC for malaria. Research will help to adapt messages and approaches to reduce audience fatigue and to promote new interventions. Malaria behaviours are not static, they change in response to new policies, interventions and messages.

Periodic national cross-sectional household surveys can provide the much-needed data on determinants of malaria behaviours, track the impact of BCC efforts, solidify and inform the evidence base, and allow us to adapt efforts to respond to a changing malaria environment. In Swaziland for example, yearly KAP studies are helping to track progress, monitor the contribution of different communication channels, and focus communication activities on the most at-risk groups [37]. Understanding how perceptions and behaviours change over time will be one of the keys to successful malaria elimination.

Investment in high-quality malaria BCC is good practice, and should be an integral component of malaria control strategies from the start. At the same time, rigorous evaluations are needed to increase the evidence base across different transmission settings. By supporting the use of BCC and research on its effectiveness, donors can be assured of a much stronger return on their investments in malaria control. If in addition to being widely available, those commodities are used properly and consistently, control or elimination of malaria becomes a more attainable goal.

## Competing interests

The authors declare that they have no competing interests.

## Authors’ contributions

HK, JK, AA drafted the article. ML, MA and FNT outlined the arguments. All authors read and approved the final manuscript.
